# Phytochemical and Pharmacological Research in *Agrimonia eupatoria* L. Herb Extract with Anti-Inflammatory and Hepatoprotective Properties

**DOI:** 10.3390/plants11182371

**Published:** 2022-09-11

**Authors:** Natalia Huzio, Andriy Grytsyk, Ain Raal, Lyubov Grytsyk, Oleh Koshovyi

**Affiliations:** 1 Department of Pharmaceutical Management, Drug Technology and Pharmacognosy, Ivano-Frankivsk National Medical University, 2 Halytska Str., 76018 Ivano-Frankivsk, Ukraine; 2 Institute of Pharmacy, Faculty of Medicine, University of Tartu, Nooruse 1, 50411 Tartu, Estonia; 3 Department of Pharmacognosy, The National University of Pharmacy, 53 Pushkinska St, 61002 Kharkiv, Ukraine

**Keywords:** *Agrimonia eupatoria* L., herb, extract, biological active substances, hepatoprotective activity

## Abstract

The most promising plant from the genus Agrimony (*Agrimonia* L.) of the *Rosaceae* family for use in medical practice is *Agrimonia eupatoria* L. Phytochemical and pharmacological research in *Agrimonia eupatoria* L. herb extract, obtained with using 40% ethanol solution as an extractant were carried out. A total of 11 free and 17 bound monosaccharides, 17 amino acids were found in the studied extract, 9 of which are essential. Gallic and ellagic acids, gallocatechin, epigallocatechin, catechin, epicatechin, and epicatechin gallate were identified in the extract of *A. eupatoria* by the HPLC method; as well as hydroxycinnamic acids: hydroxyphenylacetate, caffeic, syringic, p-coumaric, ferulic, sinapic, cinnamic and quinic acid; flavonoids: quercetin-3-D-glucoside (isoquercitrin), neohesperidin, naringenin, luteolin were found, and their quantitative content was determined, as well by spectrophotometric methods. The herb extract of *A. eupatoria* belongs to practically non-toxic substances and has pronounced anti-inflammatory (at a dose of 10.0 mg/kg anti-exudative activity reached a maximum in 5 h (88.17%)) and hepatoprotective activity (at a dose of 25 mg/kg it reduce AlAt level by 1.1 and 1.2 times, respectively; AsAt by 1.2 and 1.1 times, respectively), reduces the level of lipid peroxidation and stabilizes the membrane structures of liver cells. Thus, the herb extract of *A. eupatoria* is a promising substance for the creation of phytomedicines with anti-inflammatory and hepatoprotective activity.

## 1. Introduction

In recent years, methods of treatment using medicinal plants have become increasingly widespread. The search for promising plants in the flora of Ukraine, which are used in folk and scientific medicine and have a sufficient raw material base is an actual task of modern pharmaceutical science.

Plants of the genus Agrimony (*Agrimonia* L.) of the *Rosaceae* family, which includes 10 species that are common in the temperate zone of the Northern Hemisphere, South America, and in the mountains under the tropics, are promising objects for pharmacognostic study. In Ukraine grow 4 species of the genus Agrimony: *A. eupatoria*, *A. grandis* Andrz. L., *A. odorata* Mill. and *A. pilosa* Ledeb. [1].

The most widespread in Ukraine and used in officinal and folk practice is common agrimony (*Agrimonia eupatoria* L.). This plant is included in the State Cadaster of the Plant World of Ukraine as a priority species of medicinal plants which need scientific research [1,2].

*A. eupatoria* contains a large number of various bioactive substances: flavonoids (quercetin, rutin, hyperoside, cyanidin quercetrin, luteolin, luteolin 7-glucoside, apigenin 7-glucoside, astragalin) [3,4], hydroxycinnamic acids (caffeic, chlorogenic), terpenoids, coumarins, saponins, carbohydrates (glucose, fructose, sucrose, galactose, arabinose, rhamnose, xylose, ribose), organic acids (citric, malic, oxalic, tartaric, henna), etc.) [5,6]. The herb of *A. eupatoria* also contains nitrogen-containing compounds: choline, nicotinic acid, nicotinamide, vitamins K, PP, group B and bitters [5,7]. Samples of *A. eupatoria* raw materials grown and harvested in Ukraine were studied poorly, their research allow further comparison of the chemical composition of the raw materials according to the conditions of growth.

Today, *A. eupatoria* is used in the medicine of different countries of the world. The plant has a wide spectrum of pharmacological activity—choleretic, astringent, anti-inflammatory, antimicrobial, antiviral [8], expectorant, diuretic, hemostatic and normalizes metabolism [9,10].

In the scientific medicine of Western countries and Ukrainian folk medicine, *A. eupatoria* is used as a regulator of metabolic processes—the treatment of patients with disorders of mineral and sugar metabolism: with diabetes of various types [11,12]. Plant extracts inhibit α-glucosidase [13], have an antidiabetic effect, and play an important role in regulating glucose metabolism [12]. Korean scientists investigated its hepatoprotective effect in chronic ethanol-induced liver damage in rats. They claim that the hepatoprotective effect of *A. eupatoria* is associated with inhibition of peroxidation processes in hepatocytes [14].

Therefore, it is advisable to continue research in studying of effects of *A. eupatoria* extracts on the liver for creating new phytoremedies. In the pathogenesis of liver diseases, inflammatory and toxic effects on hepatocytes play a key role. There are several classic methods based on the use as flagene: formalin, zimazan and carrageenan to determine the anti-inflammatory activity of extracts for animals [15,16,17]. We used formalin for screening studies of the extract anti-inflammatory activity. Various toxic substances, such as alcohol, tetrachloromethane and other cytotoxic ones can be used to damage animal hepatocytes [18,19]. The choice was stopped at the use of the technique with tetrachloromethane, which was effective in the study of many other objects [18,19].

According to the data of the State Register of Medicinal Products of Ukraine [1,2], two medicinal products are registered, the composition of which includes BAS of *A. eupatoria*: *Salvat* and *Prosalad*. The rich experience of using *A. eupatoria* in folk medicine of different countries of the world and the possibilities of modern technologies and led to the creation of 12 functional and dietary supplements, which include BAS of *A. eupatoria* (Amersan, Eugastrin, Cynarosan, Novocholin, Nontusyl, Natusor Farinol, etc.) [20].

The study has a novelty and hypothesis: it was shown that a 40% (*v/v*) ethanol solution is the optimal extractant for obtaining a dry extract from the herb of *A. eupatoria* [21]. It is useful to study the chemical composition and pharmacological activity of A. eupatoria extract obtained from 40% ethanol solution to create a prospect for creating a new hepatoprotective and anti-inflammatory drug.

The aim of the current study was to research chemical composition and acute toxicity, anti-inflammatory and hepatoprotective activity of the extract of *A. eupatoria* L herb.

## 2. Results and Discussion

### 2.1. Phytochemical Research

The obtained dry herb extract of *A. eupatoria* is a loose powder of brown color with a specific smell. The yield of the extract is 18.26 ± 0.98%.

The determination of monosaccharides in the herb extract of *A. eupatoria* was carried out by the GC/MS method on chromatography Agilent 6890N/5973inert (Agilent Technologies, Santa Clara, CA, USA) on HP-5ms capillary column (30 m × 0.25 mm × 0.25 mkm, Agilent Technologies, USA) [17,22,23]. (Table 1).

In the studied extract, 11 free monosaccharides were detected, 3 were identified—D-glucose, D-galactose and D-fructose, the content of which was 120.16 mg/g, 2.82 mg/g and 116.11 mg/g, respectively.

After carrying out acid hydrolysis, the presence and quantitative content of 17 monosaccharides were determined in the herb extract of *A. eupatoria*, of which 8 were identified: D-rhamnose, L-arabinose, D-xylose, D-mannose, D-glucose, D-galactose, D-mannitol and D-dulcitol.

D-glucose dominates among the monosaccharides in the studied extract, the amount of which almost doubled after acid hydrolysis, and its content was 238.8 mg/100 g.

Quantitative determination of the content of amino acids was carried out on an amino acid analyzer AAA T-339 M (Mikrotehna, Praha, Czech Republic) in comparison with the concentration of standard amino acids [23,24]. The research was conducted on the basis of the testing center of the Institute of Livestock Breeding of the National Academy of Sciences, Kharkiv. The results of studies of the qualitative composition and quantitative content of amino acids in the herb extract of *A. eupatoria* are shown in Table 2.

According to the obtained data (Table 2), 17 amino acids, (9 of which are essential), were detected and identified in the studied sample of herb extract of *A. eupatoria*. Aspartic acid, glycine (9.03 and 8.93 mg/100 mg, respectively), alanine, valine (5.69 and 6.19 mg/100 mg, respectively) and lysine (5.03 mg/100 mg) dominate quantitatively. The quantitative content of the sum of amino acids is 7.18 g per 100 g of extract.

In our previous work [25] studying the plant *Leonurus cardiaca* L. we showed that adding amino acids to the plant extract can enhance some of its biological activity. Thus, the effect can also be influenced by the set of amino acids synthesized by the plant.

The determination of tannin components, hydroxycinnamic acids and flavonoids in the herb extract of *A. eupatoria* was carried out by the HPLC method [17] on chromatograph Agilent 1200 3D LC System Technologies (Agilent Technologies, Santa Clara, CA, USA) (Table 3).

The following substances of tannin fragments were identified by the HPLC method in the herb extract of *A. eupatoria*: two fragments of hydrolyzable tannins (gallic and ellagic acids), four simple catechins (gallocatechin, epigallocatechin, catechin and epicatechin) and one complex catechin (epicatechin gallate). The most common epicatechin and epigallocatechin were found in herb extract of *A. eupatoria*—1160 and 970 mg/100 g, respectively. By the review [9] *A. eupatoria* contains at least 2% of tannins, where 3–21% are condensed tannins, in particular proanthocyanidins are present mainly in the form of leuco-anthocyanins bioconverted by acid hydrolysis to cyanidin. Tannins have demonstrated antiseptic, astringent, antioxidant, anti-inflammatory, and anti-mutagenic properties.

**Figure 1:**
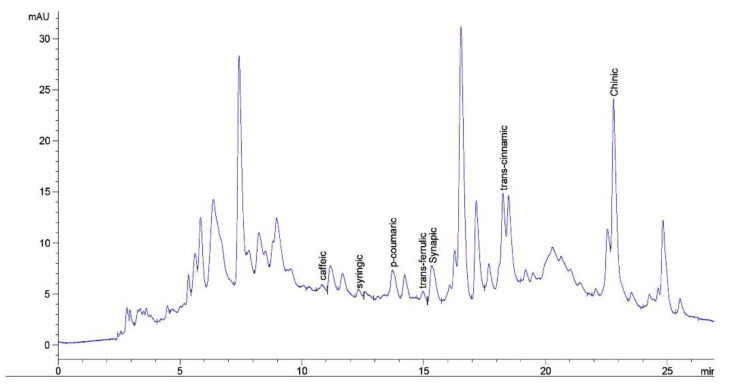
HPLC-chromatogram of hydroxycinnamic acids of the herb extract of *A. eupatoria*.

The hydroxycinnamic acids (hydroxyphenylacetate, caffeic, syringic, p-coumaric, ferulic, sinapic, cinnamic and quinic acid (69.73 mg/100 g) (Figure 1, Table 3) were identified in the extract of *A. eupatoria* grass by the HPLC method.

Caffeic acid and ellagic acid exhibit antioxidant and anti-inflammatory actions. Also, caffeic acid downregulates the inflammatory mediators nitric oxide synthase, COX-2 and TNF-α. p-coumaric acid to inhibit reactive oxygen species production, has antiangiogenic, anti-infective, antioxidant, acts as a free radical scavenger, protects pancreatic islets, prevents myocardial and lung injury. Ferulic acid has antibacterial, anti-inflammatory and other effects [9]. Hydroxyphenylacetate and p-coumaric acids predominate in the herb extract of *A. eupatoria* of the hydroxycinnamic acids. Hydroxyphenylacetate acid has anti-aggregant properties [26]. p-coumaric acid exhibits antioxidant properties, which lead to a decrease in the risk of developing stomach cancer due to a decrease in the formation of carcinogenic nitrosamines. In in vitro experiments, p-coumaric acid showed pronounced anti-inflammatory activity [27]. Also, p-coumaric acid exerts hepatoprotective and hypolipidemic effects [28].

**Figure 2:**
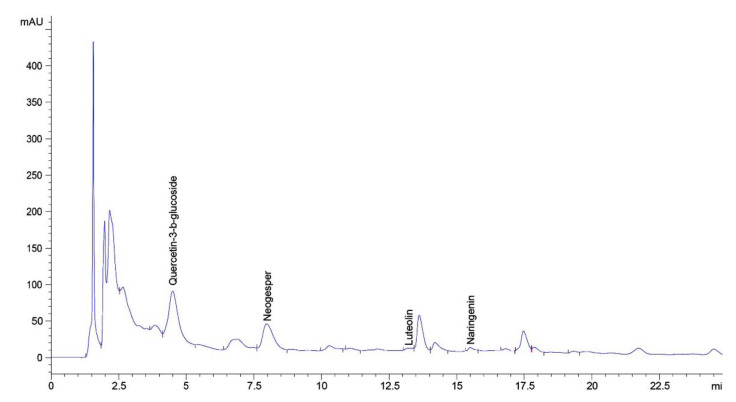
HPLC-chromatogram of the flavonoids of the herb extract of *A. eupatoria*.

As a result of the research 4 flavonoids were determined in the herb extract of *A. eupatoria* by the HPLC method (Figure 2, Table 3).

Neohesperidin, which belongs to the bitter glycosides of flavanones and provides a bitter taste of the raw material and a possible effect on the hepatobiliary system, prevails among the flavonoids in the herb extract of *A. eupatoria* [29]. This flavonoid has a sedative effect in combination with diosmin. Also, neohesperidin exhibits hypolipidemic and hypotensive activity [30]. Generally, flavonoids have a significant antioxidant, anti-inflammatory, anti-allergic potential. Kaempferol and its glycosides possess antimicrobial and analgesic properties, luteolin is a flavone reactive oxygen and nitrogen species scavenger [9,31]. The study of Karlinska et al. [4] indicated that both plants *A. eupatoria* and *A. procera* potentially good sources of polyphenols including especially flavonoid glycosides apigenin, luteolin and quercetin. Quercetin-3-D-glucoside (isoquercitrin), neohesperidin, naringenin, and luteolin were identified among the flavonoids by HPLC method in the herb extract of *A. eupatoria*.

The results of the determination of the content of the main groups of BAS of phenolic compounds in the herb extract of *A. eupatoria* by the spectrophotometric method are given in Table 2.

Thus, the obtained data will be used for the development of extract standardization methods, and the obtained extract, based on its chemical composition, is a promising substance for the development of a dosage form and the study of its anti-inflammatory and hepatoprotective activity.

### 2.2. Pharmacological Activity

#### 2.2.1. Anti-Inflammatory Activity

**Figure 3:**
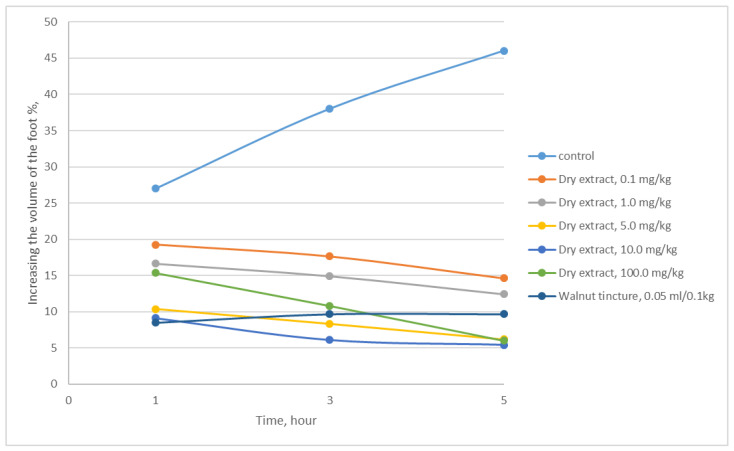
The effect of the herb extract of *A. eupatoria* on the development of limb swelling in rats.

The results of the conducted studies show that the dry herb extract of *A. eupatoria* exhibits anti-inflammatory activity on the model of formalin inflammation, which was caused by the introduction of 0.1 mL of a 2% aqueous solution of formalin under the aponeurosis of the sole of the hind paw (Table 4 and Table 5, Figure 3). Experiments were conducted on white linear male rats [32,33].

The anti-edematous effect of herb extract of *A. eupatoria* was observed already 1 h after the start of treatment, which reduced swelling by 66.19%, and it is reached a maximum in 5 h (88.17%). It was established that the most pronounced anti-exudative activity is shown by herb extract of *A. eupatoria* at a dose of 10.0 mg/kg.

*A. eupatoria* exerts an immunoprotective effect, decreases the levels of pro-inflammatory cytokines while increasing those of anti-inflammatory cytokines. It has been shown to interfere with NO regulation, stimulate the expression and activity of the antioxidant enzymes superoxide dismutase, catalase and glutathione, and to scavenge free radicals [9]. Santos et al. [31] studied the in vitro anti-inflammatory activity of both the infusion of *A. eupatoria* and the polyphenol-enriched fraction purified with n-hexane and ethyl acetate. Both extracts significantly reduced macrophage-produced NO levels in murine cell line macrophages (RAW 264.7) without adversely affecting cell viability. NO reduction was observed at concentrations of 382 µg/mL and 138 µg/mL, respectively. The anti-inflammatory and analgesic effects of water agrimony extracts devoid of a toxic effect were also documented under in vivo conditions. Ivanova et al. [34] investigated the effects of one month’s consumption of *A. eupatoria* tea in healthy volunteers. The results indicate that the tea has potential in improving markers of lipid metabolism, oxidative status and inflammation. Triterpenoid saponins not studied by us show anti-inflammatory, antioxidant and antimicrobial effects [9]. Also in traditional medicine the plant has long been used to treat lung inflammation, inflammatory conditions of the oral mucosa, and liver diseases [9].

#### 2.2.2. Acute Toxicity and Hepatoprotective Activity

The study of acute toxicity showed that the herb extract of *A. eupatoria* belongs to practically non-toxic substances when it is administered intragastrically (LD_50_ > 5000 mg/kg). As a result of the conducted research, it was established that intragastric administration of the herb extract of *A. eupatoria* in doses of 8000–10,000 mg/kg does not lead to the death of animals, changes in the morphological structure of the internal organs of the experimental animals do not occur, which indicates the absence of toxic effects of the extract, and characterizes them as practically non-toxic (toxicity class V) according to the classification of substances by toxicity [32]. According to the recommendations of the State expert center of the Ministry of Health of Ukraine, it is impossible to establish the average lethal dose of the drug in this case [32].

The results of the study of the hepatoprotective properties of herb extract of *A. eupatoria* on the model of acute tetrachloromethane hepatitis [19,32] are shown in Table 6.

The conducted studies showed that during the development of model toxic hepatitis, a significant increase in the level of 4.5-fold general and 5.2-fold ascorbate-dependent lipid peroxidation was observed relative to intact animals. The destruction of hepatocyte membranes is evidenced by a 4.8-fold and 2.4-fold increase in the activity of AlAt, and AsAt enzymes, which is veracious in intact animals.

The extract of *A. eupatoria* at a dose of 25 mg/kg and the reference drug *Silibor* veraciously reduced the level of general and ascorbate-dependent lipid peroxidation relative to the hepatitis model. The extract of *A. eupatoria* and *Silibor* are relatively active (reduction of AlAt, level by 1.1 and 1.2 times, respectively; AsAt by 1.2 and 1.1 times, respectively), in terms of the level of inhibition of cytolytic processes.

In the liver, the herb of *A. eupatoria* inhibits TLR-4 signaling and helps alleviate liver injury. The aqueous extracts prepared from the aerial parts (stems and leaves) of *A. eupatoria* and *A. pilosa* inhibited hepatitis surface antigen secretion [9]. The hepatoprotective effect of *A. eupatoria* aqueous extract has also been proven by a clinical trial on 80 subjects with elevated alanine transaminase levels in a randomized, double-blind, placebo-controlled, 8-week study. The extract has been shown to protect against liver injury due to its lipid lowering and antioxidant activities [35]. The effect of *A. eupatoria* on liver tissue appears to be even broader: Kwon et al. [36] suggest that the aqueous extract prepared from the aerial parts of *A. eupatoria* and some other plants of the genus *Agrimonia* contain potential antiviral activity against hepatitis B virus.

Thus, the obtained results indicate that the dry herb extract of *A. eupatoria* has a pronounced hepatoprotective effect, reduces the level of lipid peroxidation and stabilizes the membrane structures of liver cells.

## 3. Materials and Methods

### 3.1. Chemicals and General Experiments

Deionized water was produced using Millipore Simplicty UV station (Merck Millipore, Burlington, MA, USA). Acetonitrile, formic acid, pyridine, ethanol, methanol, hydroxylamine hydrogen chloride, acetic anhydride, dichlorethane, hydrochloric acid, heptane, ethyl acetate, formalin, tetrachlormethane was purchased from VWR (Radnor, PA, USA). Chlorogenic acid, rutin, pyrogallol were purchased from Carl Roth (Karlsruhe, Germany). Hydroxyphenylacetate, caffeic, syringic, p-coumaric, ferulic, sinapic, cinnamic, quinic acids and rutin, quercetin-3-D-glucoside (isoquercitrin), naringin, neohesperidin, quercetin, naringenin, kaempferol, sorbitol, luteolin and aluminum chloride were purchased from Sigma-Aldrich (Sant Louis, MI, USA).

### 3.2. Plant Material

The starting raw material for obtaining the dry extract was chopped grass of the *A. eupatoria*, which was harvested in 2019 during the mass flowering of plants in the vicinity of the village Vovchyntsi, Tysmenetska district, Ivano-Frankivsk region (48.95890196269463, 24.753323924067537). About 1 kg of plant material (15–20 cm of the upper parts of the flowering plants) was collected from the same growing place. The identity of the plant was established by the consulting assistance of Professor A.R. Grytsyk from the Ivano-Frankivsk National Medical University (IFNMU) according to the botanical catalog [37]. Voucher specimens No. 423–428 were deposited at the Department of Pharmaceutical Management, Drug Technology and Pharmacognosy, Ivano-Frankivsk National Medical University. The raw material was dried for 10 days at room temperature in a well-ventilated area for ten days and stored in paper bags.

### 3.3. Preparation of the Extract

The object of the study was the dry extract of *A. eupatoria.* 50 g of *A. eupatoria* dried herb was ground to a particle size of 0.5–3 mm and loss on drying was tested [38,39,40]. The plant material was placed in a flask, poured into 750 mL of a 40% (*v/v*) solution of ethanol, heated under reflux in a water bath for 30 min and extracted for 4 h at room temperature. After isolation of the extract, a new portion of the same extractant (750 mL) was added to the waste and repeated extraction was carried out under the same conditions. The obtained liquid extract was dried in a dry oven at the temperature of 50–60 °C to a moisture content of no more than 5% [38].

### 3.4. Phytochemical Research

#### 3.4.1. Monosaccharides

Determination of free monosaccharides was carried out by the method of gas-liquid chromatography-mass spectrometry on chromatography (GC/MS) Agilent 6890N/5973 inert (Agilent Technologies, Santa Clara, CA, USA). HP-5ms capillary column (30 m × 0.25 mm × 0.25 mkm, Agilent Technologies, Santa Clara, CA, USA). The evaporator temperature is 250 °C, the interface temperature is 280 °C. The separation was carried out in the temperature programming mode—the initial temperature of 160 °C was maintained for 8 min, then it was raised with a gradient of 5 °C/min to 240 °C. The final temperature was maintained for 6 min. The sample with a volume of 1 μL was injected in the mode of flow division 1:50. Detection was carried out in SCAN mode in the range (38–400 *m/z*). The carrier gas flow rate through the column is 1.2 μL/min [17,23].

Sample preparation and extract analysis. A 50 mg portion of the extract was placed in a round-bottomed flask, and 5.0 mL of a solution of 80% ethanol P (*v/v*) with an internal standard (sorbitol) was added at the rate of 500 μg per sample. Extraction of free monosaccharides was carried out in a water bath at 100 °C using a reflux condenser for 2 h. To obtain aldonitrile monosaccharides derivatives was taken 2 mL of the extract, evaporated to dryness on a rotary evaporator, and 0.3 mL of derivatizing reagent (32 mg/mL of hydroxylamine hydrogen chloride in a mixture of pyridine/methanol (4:1 *v/v*)) was added. The dissolved extract was kept for 25 min at 75 °C. 1 mL of acetic anhydride was added and kept for 15 min at 75 °C for acetylation of aldonitrile monosaccharides derivatives. 2 mL of dichloroethane was added to the reaction mixture, and the excess of derivatization reagents was removed by double extraction with a 1N solution of hydrochloric acid and purified water. The dichloroethane layer was dried to dryness and dissolved in 300 μL of a heptane/ethyl acetate mixture (1:1 *v/v*).

Identification of monosaccharides of the studied mixture was performed by comparing the retention times of standard monosaccharides and using the NIST 02 mass spectrum library. Quantitative analysis was performed by adding a solution of the internal standard to the tested samples. Sorbitol solution was used as an internal standard.

The mass of monosaccharides per 100 g of raw material in μg was calculated according to the formula:

(1) $$X=\frac{S_{x}\times \mathrm{Mvn}.\mathrm{st}.\times 100}{\mathrm{Spa}.\mathrm{is}\times m}$$

where: *S_x_* is the peak area of the studied monosaccharide; Mvn.st.—mass of the internal standard per sample; Spa.is.—the peak area of the internal standard; *m* is the weight of the raw material, in grams. Three technological repetitions were carried out.

#### 3.4.2. Amino Acids

Determination of amino acids was carried out on an amino acid analyzer AAA T-339 M (Mikrotehna, Praha, Czech Republic) [23,24].

Sample preparation and extract analysis. The dried weight extract (100 μg) was placed in a test tube for hydrolysis and 5 mL of purified water was added, mixed, and an equal amount of concentrated hydrochloric acid was added. Hydrolysis was carried out at a temperature of 120 °C for 15 min. After that, the sample was neutralized with dry sodium hydroxide to pH 11, transferred to a porcelain cup for 1 h in order to accelerate the evaporation of ammonia. The solution of hydrochloric acid was added to set the pH to 2.2. The sample was filtered. 0.1–0.5 mL of liquid was taken, after it was brought to a volume of 2 mL with a buffer solution. A sample volume is 50 μL.

Identification and quantification of amino acids was carried out in the hydrolysate of the extract in comparison with the concentration of standard amino acids. Three technological repetitions were carried out.

#### 3.4.3. Tannin Fragments

The qualitative composition and quantitative content of tannin fragments in the extract was determined by HPLC on an Agilent 1200 3D LC System Technologies chromatograph (Agilent Technologies, Santa Clara, CA, USA) [17,41,42].

As a mobile phase, solvent A was used, which is 0.1% trifluoroacetic acid, 5% acetonitrile and purified water P, the pH of the solution is 2.08, and solvent B is 0.1% trifluoroacetic acid and acetonitrile. Chromatography mode is maximum flow rate of mobile phase 0.1 mL/min, which is the maximum working pressure of eluent 400 bar (40 kPa); the temperature of the column thermostat is 25 °C; the volume of the injected sample is 5–20 μL, the chromatography time is 40 min. Elution-gradient: 0 min 100% “B”, 8 min 12% “B”, 10 min 12% “B”, 15 min 25% “B”, 20 min 25% “B”, 25 min 75% “B”, 28 min 75%, 29 min 100%. The scanning time is 0.6 s, the detection range is 190—400 nm, the wavelength is 280, 255 nm.

Sample preparation was carried out as follows: the chopped MPM was weighed with a mass of 100.0 mg (accurate measurement), extracted 50.0 mL of 95% methanol solution in an ultrasonic bath at 80 KHz and 45 °C for 30 min. The extract was cooled and filtered, the filtrate was evaporated at 50 °C in a rotary evaporator. The dry residue was filtered through a membrane filter with a pore diameter of 0.45 μm in 100 mL of mobile phase A before chromatography. Three technological repetitions were carried out.

#### 3.4.4. Hydroxycinnamic Acids and Flavonoids

The qualitative composition and quantitative content of flavonoids and hydroxycinnamic acids were determined by HPLC on an Agilent 1200 chromatograph (Agilent Technologies, Santa Clara, CA, USA) [43,44,45,46].

Acetonitrile (eluent A) and a 0.1% solution of formic acid in water (eluent B) were used as mobile phases. Separation was performed on a ZorbaxSB-C18 (for flavonoids), Zorbax SB-Aq (for hydroxycinnamic acids) (3.5 μm, 150 mm × 4.6 mm) column (Agilent Technologies, Santa Clara, CA, USA). Chromatography mode is carrier gas flow rate through the column 0.25 mL/min, thermostat temperature 30 °C, injection volume 4 μL. Elution was performed in gradient mode (Table 7).

Preparation of samples for analysis was 50 mg (accurate measurement) of the extract was dissolved in 5 mL of 60% (*v/v*) (for hydroxycinnamic acids) or 70% (*v/v*) (for flavonoids) in ethanol solution in an ultrasonic bath at 80 °C for 1 h. The experiment was conducted in glass hermetic vials with teflon caps. The obtained extract was centrifuged at 3000 rpm and it was filtered through disposable membrane filters with pores of 0.22 μm.

Detection was carried out using a diode-matrix detector with signal registration at wavelengths of 250, 275 nm (for hydroxycinnamic acids) and 280, 365 nm (for flavonoids) and fixation of absorption spectra in the range of 210–700 nm.

Identification and quantitative analysis were carried out using standard solutions of phenolic compounds: hydroxyphenylacetate, chlorogenic, caffeic, syringic, p-coumaric, ferulic, sinapic, cinnamic, quinic acids and rutin, quercetin-3-D-glucoside (isoquercitrin), naringin, neohesperidin, quercetin, naringenin, kaempferol and luteolin.

The content of compounds (X) in mg/100 g is determined by the formula:

(2) $$X=\frac{C\times V}{m}$$

where: C is the concentration of the compound, determined chromatographically, in mg/mL; V is volume of extract, in ml; m is the mass of the studied extract, in grams. Three technological repetitions were carried out.

#### 3.4.5. Spectrophotometry Methods

##### Determination of the Quantitative Content of Total Flavonoids

The quantitative determination of the number of flavonoids per rutin was determined by a method based on the complexation reaction of flavonoids with aluminum chloride. The maximum absorption of the flavonoid complex was observed by aluminum chloride at a wavelength of 410 nm [25,38]. 6–7 technological repetitions were carried out.

100.0 mg (exact weight) of the extract was placed in a 100.0 mL round-bottomed flask and solved in 70% ethanol (*v/v*) in a ratio of 1:30 in a reflux water bath for 30 min at the boiling temperature of the solvent. The extract was filtered into a 100.0 mL volumetric flask and brought up to the mark with the extractant and mixed.

1.0 mL of the extract, 1.0 mL of 2% aluminum chloride solution (*m/v*) in 96% ethanol, 2 drops of glacial acetic acid was added in a round-bottom flask with a capacity of 25.0 mL. The volume of the solution was brought up to the mark with 96% ethanol. The optical density of the obtained solution was measured on Specol 1500 spectrophotometer (Neuchâtel, Switzerland) at a wavelength of 410 nm in a cuvette with a layer thickness of 10 mm.

The solution contained 1.0 mL of the extract, two drops of glacial acetic acid and adjusted to the mark with 96% ethanol in a 25.0 mL volumetric flask was used for comparison. In parallel, under the same conditions, the optical density of a solution containing 1.0 mL of a 0.05% solution of a standard sample of rutin (*m/v*), which was prepared similarly to the solution under study, was measured.

The content of flavonoids in terms of rutin and absolutely dry raw materials was calculated according to formula (3):

(3) $$X=\frac{A1\times m0\times 1\times 100\times 25\times 100\times 100}{A0\times m\times 100\times 3\times 25\times \left(100-W\right)}$$

where: A1 is the optical density of the investigated solution; m0 is the weight of a standard sample of rutin, in grams; A0 is the optical density of the rutin solution of the standard sample; m_1_—raw material weight, in grams; W—mass loss during drying of the extract, in %.

Preparation of a solution of a rutin standard sample: 0.05 g (precisely weighed) of standard rutin (FS 42-2508-87), dried to a constant weight at a temperature of 130–135 °C, was dissolved in a 100 mL volumetric flask in a small amount 96% of ethanol when heated in a boiling water bath, cooled and the volume of the solution was brought up to the mark with the same ethanol (1 mL of the standard sample solution contains 0.0005 g of rutin).

##### Determination of the Quantitative Content of Hydroxycinnamic Acid Derivatives

The content of hydroxycinnamic acid derivatives per chlorogenic acid in the extract was determined by spectrophotometry [31,47]. 6–7 technological repetitions were carried out.

100.0 mg (exact weight) of the extract was dissolved in 20% ethanol solution (*v/v*) in a 200 mL flask. The solution is filtered, the filtrate is quantitatively transferred to a 250.0 mL volumetric flask and the volume is brought up to the mark with the same solvent.

1 mL of the extract solution is added to a 25.0 mL volumetric flask and the volume of the solution is brought up to the mark with a 20% ethanol solution (*v/v*). The optical density of the obtained solution was measured on a spectrophotometer at a wavelength of 327 nm. 20% ethanol solution (*v/v*) was used as a comparison solution.

The content of the sum of hydroxycinnamic acids in terms of chlorogenic acid in percent was calculated according to formula (4):

(4) $$X=\frac{A\times 250\times 25\times 100}{531\times m\times 1\times \left(100-W\right)}\;,$$

where: A is the optical density of the studied solution; m—mass of raw material, in grams; 531—specific index of absorption of chlorogenic acid; W—mass loss during drying of the extract, in %.

##### Determination of the Quantitative Content of Tannins

The quantitative content of tannins per pyrogallol was determined by the method of SPhU 2.1 [17,38]. 6–7 technological repetitions were carried out.

100.0 mg (exact weight) of the extract is placed in a round-bottom flask with a capacity of 250 mL, solved in water and the volume of the solution is brought to 250 mL with water, filtrate through filter paper with a diameter of 125 mm. Discard the first 25 mL of filtrate.

5.0 mL of the extract solution is brought to 25.0 mL with water. A mixture of 2.0 mL of the obtained solution, 1.0 mL of phosphorus-molybdenum-tungsten reagent and 10.0 mL of water is adjusted to a volume of 25.0 mL with a solution of 290 g/L of sodium carbonate. After 30 min, measure the optical density of the solution at a wavelength of 760 nm (A1), using water as a compensating solution.

0.10 g of skin powder standard sample is added to 10 mL of filtrate and vigorously shaken for 60 min. The mixture is filtered and 5.0 mL of the filtrate is made up to a volume of 25.0 mL with water.

A mixture of 2.0 mL of the obtained solution, 1.0 mL of phosphorus-molybdenum-tungsten reagent and 10.0 mL of water is adjusted to a volume of 25.0 mL with a solution of 290 g/L of sodium carbonate. After 30 min, measure the optical density of the solution at a wavelength of 760 nm (A2), using water as a compensating solution.

##### Standard Solution

Immediately before the measurement, 50.0 mg of pyrohalol is dissolved in water R and the volume of the solution is adjusted to 100.0 mL with the same solvent. 5.0 mL of the resulting solution is brought to a volume of 100.0 mL with water P.

A mixture of 2.0 mL of the obtained solution, 1.0 mL of phosphorus-molybdenum-tungsten reagent P and 10.0 mL of water P is adjusted to a volume of 25.0 mL with a solution of 290 g/L of sodium carbonate P. After 30 min, measure the optical density (2.2.25) of the solution at a wavelength of 760 nm (A3), using water R as a compensating solution.

The content of tannins, in terms of pyrogallol, in percent, is calculated by the formula (5):

(5) $$X=\frac{62.5\times \left(A1-A2\right)\times m2}{A3\times m1\times 100}\;,$$

where m1 is the mass of the tested sample, g; m2 is the mass of pyrogallol, g.

### 3.5. Pharmacological Activity

The study of the pharmacological activities of the *A. eupatoria* herb extract was carried out with the advisory assistance of different experts in the field. Experimental work was carried out in the scope of simple pharmacological screening [32]. The experiments were carried out on white nonlinear mice and Wistar rats, bred in the IFNMU vivarium, which were standardized by physiological and biochemical parameters and were kept under vivarium conditions in accordance with hygiene standards. All subjects gave their informed consent for inclusion before they participated in the study. The study was conducted in accordance with the Declaration of Helsinki, and the protocol was approved by the Ethics Committee of Ethics Commission of Ivano-Frankivsk Medical University (Protocol No. 114/20 dated 21 May 2020, “Studies of some wild and cultivated medicinal plants of the western region of Ukraine and development drugs based on them”, No state registration 0110U006205). The experiment was carried out in accordance to the International Principles of the European Convention for the Protection of Vertebrate Animals Used for Experimental and Other Scientific Purposes [48,49,50].

#### 3.5.1. Anti-Inflammatory Activity

The study of the anti-inflammatory activity of the herb extract of *A. eupatoria* was carried out on the model of formalin inflammation, which was caused by the introduction of 0.1 mL of a 2% (*m/v*) aqueous solution of formalin under the aponeurosis of the sole of the hind paw. Experiments were conducted on white linear male rats weighing 180–220 g [32,33].

The animals were divided into 7 groups of 6 animals each. The first group—control animals, which were injected with 0.1 mL of 2% (*m/v*) aqueous formalin solution. The animals of groups 2–6 were orally administered a herb extract of *A. eupatoria* in 2 h and immediately after the introduction of the phlogogenic agent in the appropriate doses. The seventh group of animals was injected with a reference substance with a known anti-inflammatory effect—walnut (*Juglans regia* L.) tincture in a dose of 0.05 mL per 100 g of animal body weight.

The volume of the paw was measured with an oncometer before the start of the experiment, after 1 h, after 3 h, and at the time of the greatest development of swelling after 5 h.

The effect of extracts of *A. eupatoria* was evaluated by their ability to inhibit paw swelling in rats. The anti-inflammatory efficiency was calculated according to the formula:

$$X=\frac{\left(V_{k}-V_{0}\right)\times 100}{V_{k}}\;,$$

where: V_k_ is the average increase in the volume of the swollen paw in the control; V_0_ is the average increase in the volume of the swollen paw in treated animals.

#### 3.5.2. Acute Toxicity and Hepatoprotective Activity

The study of acute toxicity is mandatory in the complex of preclinical studies of new medicines. The method of preclinical study of the harmlessness of medicines was used to study the acute toxicity of the herb dry extract of the *A. eupatoria* [32,51].

The research was conducted on white outbred mice of both sexes, which were obtained from the vivarium of IFNMU, and have weight 18–22 g, which were on a regular diet. In the experiment, a group of 6 animals was used, which were injected with an aqueous solution of the extract of the *A. eupatoria* and a control group. The solutions were administered intragastrically with the help of a metal probe in increasing doses.

The animals were observed for 14 days. The effect of the extract was evaluated by integral indicators (general condition, changes in body position, skin condition, color of mucous membranes, body temperature) and individual symptoms (diarrhea, drowsiness, tremors, convulsions, etc.).

The study of the hepatoprotective activity of the herb extract of *A. eupatoria* was carried out on the model of acute tetrachloromethane hepatitis [19,32]. Experiments were conducted on white male rats weighing 200–240 g, divided into 6 groups of 6–9 animals. Liver damage was caused in animals of the first to fifth groups by a 50% oil solution of tetrachloromethane in a dose of 0.8 mL per 100 g of animal weight for 2 days with an interval of 24 h. The investigated extract and the comparison drug were administered to the animals 1 h and 2 h after the administration of the hepatotropic poison.

The animals of groups 2–4 were injected with an aqueous solution of the herb extract of A. eupatoria in doses of 50, 25, and 10 mg per 1.0 kg of the animal’s weight, respectively. Animals of the fifth group were administered the comparative drug “Silibor” in a dose of 25 mg per 1.0 kg of animal weight. The sixth group is intact animals (IA).

Rats were decapitated on the third day after the first injection of tetrachloromethane. The conclusion about the pharmacotherapeutic effectiveness of the studied extract was made on the basis of biochemical and functional indicators of the state of the liver, which were determined in 24 h after the last injection of tetrachloroethane. The total level of lipid peroxidation (TLoP) was determined in liver homogenates according to the method of L. Ernster, and ascorbate-dependent lipid peroxidation (ADL)—according to the method of L. Ernster in the modification of A.I. Archakova, Yu.V. Vladimirova. The activity of cytolysis enzymes—alanine aminotransferase (AlAt) and aspartate aminotransferase (AsAt) was determined in blood serum using the Reitman-Frenkel method [32,51], which reflect the state of cell membranes of hepatocytes.

### 3.6. Statistical Analysis

Statistical properties of random variables with n-dimensional normal distribution are given by their correlation matrices, which can be calculated from the original matrices. Statistical assessment all pharmacological data are reported as mean ± SEM and were analyzed using STATISTICA 6 software with one-way ANOVA. *p* values less than 0.05 were assumed to be statistically significant [38,52,53].

## 4. Conclusions

As a result of phytochemical and pharmacological studies, it has been shown that the herb extract of *A. eupatoria* is a promising substance for the creation of drugs with anti-inflammatory and hepatoprotective activity.

## Figures and Tables

**Table 1:** Qualitative composition and quantitative content of monosaccharides in the herb extract of *A. eupatoria*.

Name of Monosaccharide	Monosaccharide Content, mg/100 g	
	Free	After Acid Hydrolysis
D-Rhamnose		24.42 ± 0.27
L-arabinose		2.08 ± 0.03
D-Xylose		52.98 ± 1.17
D-Mannose		10.32 ± 0.27
D-Glucose	120.16 ± 1.63	238.8 ± 2.02
D-Galactose	2.82 ± 0.04	74.97 ± 2.01
D-Mannitol		4.49 ± 0.07
Sorbitol	internal standard	
D-Fructose	116.11	
D-Dulcitol		4.00 ± 0.05

**Table 2:** The content of amino acids in the herb extract of *A. eupatoria*.

Amino Acid	Content, mg/100 g	Amino Acid	Content, mg/100 g
Aspartic acid	903 ± 21	Methionine	311 ± 9
Threonine	309 ± 9	Isoleucine	426 ± 12
Serine	621 ± 18	Leucine	461 ± 9
Glutamic acid	115 ± 3	Tyrosine	213 ± 5
Proline	472 ± 11	Phenylalanine	323 ± 11
Cystine	82 ± 2	Histidine	213 ± 7
Glycine	893 ± 27	Lysine	503 ± 10
Alanine	569 ± 22	Arginine	155 ± 4
Valin	619 ± 7		

**Table 3:** Qualitative composition and quantitative content of phenolic compounds in the herb extract of *A. eupatoria*.

The Name of the Substance	Quantitative Content, mg/100 g
Tannin components	
Gallic acid	8.0 ± 0.3
Gallocatechin	210.0 ± 4.2
Epigallocatechin	970.0 ± 9.2
Catechin	380.0 ± 7.6
Epicatechin	1160.0 ± 12.7
Epicatechin gallate	630.0 ± 9.4
Ellagic acid	7 ± 0.02
Hydroxycinnamic acids	
Hydroxyphenylacetate	916.5 ± 11.4
Caffeic acid	552.4 ± 10.3
Syringic acid	172.6 ± 3.5
*p*-Coumaric acid	82.7 ± 0.9
Ferulic acid	738.8 ± 13.3
Synapic acid	381.3 ± 7.2
Cynamic acid	225.2 ± 4.1
Flavonoids	
Isoquercitrin	916.7 ± 10.7
Neohesperidin	3850.9 ± 34.5
Naringenin	308.2 ± 5.2
Luteolin	332.1 ± 6.1
**BAS group**	**Contents of BAS, %, ** $\overset{-}{x}\pm \Delta \overset{-}{x}$ **, n = 6**
Hydroxycinnamic acids	6.21 ± 0.11
Flavonoids	10.20 ± 0.33
Tannins	17.16 ±0.37

**Table 4:** The effect of the herb extract of *A. eupatoria* on the development of limb swelling in rats.

Groups	Conditional Designation of the Drug	Dose, %	Increasing the Volume of the Foot, %, $\overset{-}{x}\pm \Delta \overset{-}{x}$		
			After 1 h	After 3 h	After 5 h
1	Control		27.00 ± 0.33	38.0 ± 0.28	46.00 ± 0.36
2	Dry extract of *A. eupatoria*	0.1 mg/kg	19.27 ± 0.23 *	17.64 ± 0.05	14.63 ± 0.05 *
3		1.0 mg/kg	16.66 ± 0.29 *	14.94 ± 0.04	12.45 ± 0.03
4		5.0 mg/kg	10.37 ± 0.08 *	8.35 ± 0.06	6.22 ± 0.05 *
5		10.0 mg/kg	9.13 ± 0.11 *	6.11 ± 0.07 *	5.44 ± 0.05 *
6		100.0 mg/kg	15.36 ± 0.11 *	10.82 ± 0.04 *	6.00 ± 0.07 *
7	Walnut tincture	0.05 mL/0.1 kg	8.51 ± 0.04 *	9.68 ± 0.03	9.68 ± 0.04 *

Note. * reliability of deviations in relation to the data of the control group (*p* ≤ 0.05).

**Table 5:** Anti-exudative activity of the herb extract of *A. eupatoria*.

Groups	Drug	Dose	Inhibition Index of the Inflammatory Reaction, %		
			After 1 h	After 3 h	After 5 h
2	The dry extract of *A. eupatoria*	0.1 mg/kg	28.63	53.58	68.17
3		1.0 mg/kg	38.30	60.68	72.96
4		5.0 mg/kg	61.59	78.03	86.48
5		10.0 mg/kg	66.19	83.92	88.17
6		100.0 mg/kg	43.11	71.52	86.96
7	Walnut tincture	0.05 mL/0.1 kg	68.48	74.53	78.96

**Table 6:** The results of the study of the hepatoprotective activity of herb extract of *A. eupatoria*.

Group	Object of the Study	AlAt, mmol/L	AsAt, mmol/L	LP, mmol/h*mL	ALP, mmol/h*mL
1	Control	5.22 ± 0.07 *	4.07 ± 0.07 *	257.55 ± 27.86 *	789.91 ± 31.48 *
2	Extract, 50 mg/kg	5.12 ± 0.16 *	4.26 ± 0.19 *	97.58 ± 16.81 */**	414.04 ± 24.16 */**
3	Extract, 25 mg/kg	4.78 ± 0.31 *	3.31 ± 0.75 *	230.77 ± 13.62 *	341.14 ± 8.46 *
4	Extract, 10 mg/kg	2.81 ± 0.14 */**	2.41 ± 0.29 */**	120.75 ± 7.86 */**	359.85 ± 5.94 */**
5	Silibor	4.27 ± 0.25 */**	3.56 ± 0.12 */**	105.16 ± 16.94 */**	374.18 ± 45.48 */**
6	Intact animals	1.08 ± 0.12	1.68 ± 0.19	57.29 ± 3.41	149.80 ± 11.34

Notes: *—in relation to intact animals, *p* ≤ 0.05; **—veracious relation to the hepatitis model, *p* ≤ 0.05. AlAt—alanine aminotransferase; AsAt—aspartate aminotransferase; LP—lipid peroxidation; ALP—ascorbate-dependent lipid peroxidation.

**Table 7:** Parameters of the gradient mode of elution of hydroxycinnamic acids and flavonoids.

Hydroxycinnamic Acids				
Time, min	0	20	27	35
Eluent A, % (*v/v*)	25	75	100	100
Eluent B, % (*v/v*)	75	25	0	0
Flavonoids				
Time, min	0	20	22	30
Eluent A, % (*v/v*)	30	70	100	100
Eluent B, % (*v/v*)	70	30	0	0
